# Physical activity and risk of cancers of the colon and rectum: an Italian case-control study

**DOI:** 10.1038/sj.bjc.6690304

**Published:** 1999-04

**Authors:** A Tavani, C Braga, C La Vecchia, E Conti, R Filiberti, M Montella, D Amadori, A Russo, S Franceschi

**Affiliations:** Istituto di Ricerche Farmacologiche ‘Mario Negri’, Via Eritrea 62, Milan, 20157, Italy; Istituto di Statistica Medica e Biometria, Università degli Studi di Milano, Via Venezian 1, Milan, 20133, Italy; Istituto Regina Elena per lo Studio e la Cura dei Tumori, Via Regina Elena 299, Roma, 00100, Italy; Istituto Nazionale per la Ricerca sul Cancro, Viale Benedetto XV 10, Genova, 16132, Italy; Istituto per lo Studio e la Cura dei Tumori ‘Senatore Pascale’, Napoli, 80100, Italy; Istituto Oncologico Romagnolo, Ospedale Pierantoni, Via Forlanini, Forlì, 47100, Italy; Centro di Riferimento Oncologico, Via Pedemontana Occ.le, Aviano (Pordenone), 33081, Italy

**Keywords:** case-control study, colon cancer, epidemiology, physical activity, rectal cancer

## Abstract

We investigated the relationships between risk of colon and rectal cancers and physical activity in both sexes at different ages by a case-control study conducted between 1991 and 1996 in six Italian centres. Cases were 1225 patients (688 men, 537 women) below the age of 75 with colon cancer and the controls included 4154 patients (2073 men, 2081 women) admitted to hospital for acute, non-neoplastic conditions. We also analysed 722 cases of rectal cancer. Compared with the lowest level of occupational physical activity at 30–39 years old the odds ratios (OR) for the highest level were 0.64 (95% confidence interval, CI 0.44–0.93) in men and 0.49 (95% CI 0.33–0.72) in women. The inverse association in both sexes was similar at 15–19 and 50–59 years old. No association was found in either sex for leisure-time physical activity. For both sexes the inverse relationship between occupational physical activity at 30–39 years old and colon cancer risk was not significantly heterogeneous across strata of selected covariates, and for ascending, transverse, descending and sigmoid colon. Rectal cancer risk was not associated with any measure of physical activity (OR = 1.32 for men and 0.88 for women for the highest level of occupational physical activity at 30–39 years old compared with the lowest). This study confirms that occupational physical activity is protective against colon, but not against rectal cancer. © 1999 Cancer Research Campaign


*British Journal of Cancer* (1999) **79**(11/12), 1912–1916
© 1999 Cancer Research Campaign
Article no. bjoc.1998.0304

### Physical activity and risk of cancers of the colon and rectum: an Italian case-control study

**A Tavani[1], C Braga[1], C La Vecchia[1,2], E Conti[3], R Filiberti[4], M Montella[5], D Amadori[6], A Russo[7] and S Franceschi[7]**

[1]Istituto di Ricerche Farmacologiche ‘Mario Negri’, Via Eritrea 62, 20157 Milan, Italy; [2]Istituto di Statistica Medica e Biometria, Università degli Studi di Milano, Via Venezian 1, 20133 Milan, Italy; [3]Istituto Regina Elena per lo Studio e la Cura dei Tumori, Via Regina Elena 299, 00100 Roma, Italy; [4]Istituto Nazionale per la Ricerca sul Cancro, Viale Benedetto XV 10, 16132 Genova, Italy; [5]Istituto per lo Studio e la Cura dei Tumori ‘Senatore Pascale’, 80100 Napoli, Italy; [6]Istituto Oncologico Romagnolo, Ospedale Pierantoni, Via Forlanini, 47100 Forlì, Italy; and [7]Centro di Riferimento Oncologico, Via Pedemontana Occ.le, 33081 Aviano (Pordenone), Italy

**Summary** We investigated the relationships between risk of colon and rectal cancers and physical activity in both sexes at different ages by a case-control study conducted between 1991 and 1996 in six Italian centres. Cases were 1225 patients (688 men, 537 women) below the age of 75 with colon cancer and the controls included 4154 patients (2073 men, 2081 women) admitted to hospital for acute, non-neoplastic conditions. We also analysed 722 cases of rectal cancer. Compared with the lowest level of occupational physical activity at 30–39 years old the odds ratios (OR) for the highest level were 0.64 (95% confidence interval, CI 0.44–0.93) in men and 0.49 (95% CI 0.33–0.72) in women. The inverse association in both sexes was similar at 15–19 and 50–59 years old. No association was found in either sex for leisure-time physical activity. For both sexes the inverse relationship between occupational physical activity at 30–39 years old and colon cancer risk was not significantly heterogeneous across strata of selected covariates, and for ascending, transverse, descending and sigmoid colon. Rectal cancer risk was not associated with any measure of physical activity (OR = 1.32 for men and 0.88 for women for the highest level of occupational physical activity at 30–39 years old compared with the lowest). This study confirms that occupational physical activity is protective against colon, but not against rectal cancer.

**Keywords:** case-control study; colon cancer; epidemiology; physical activity; rectal cancer

Most cohort and case-control studies have found an inverse association between physical activity and colon cancer risk usually similar in either sex and any colon anatomical subsite (Macfarlane and Lowenfels, 1994; Longnecker et al, 1995; Colditz et al, 1997; Slattery et al, 1997). There is also general agreement on a substantial lack of association between physical activity and risk of rectal cancer both in prospective and case-control studies (Macfarlane and Lowenfels, 1994; Colditz et al, 1997). Scanty data are available on the age at which physical activity effects the greatest benefit. Most studies on physical activity at work considered the usual lifetime occupation, while other studies have examined specific times prior to diagnosis. A prospective study of Wisconsin women (Marcus et al, 1994) found that those who reported strenuous physical activity from 14 to 22 years old were at a similar risk of colon cancer to those reporting no activity. Another prospective study on Harvard alumni (Lee et al, 1991) showed that those with high levels of activity throughout their lives were at the lowest risk of developing colon cancer. Similarly, a case-control study from North America showed that a high level of vigorous leisure time physical activity during the past 20 years was protective against colon cancer in both men and women (Slattery et al, 1997).

However, as most findings relate to men and to North American and North European populations, further studies are needed in other populations, especially in women, to establish the ages at which physical activity has most impact, and to assess whether the protection is restricted to any specific anatomical subsites of the large bowel. To examine these issues we analysed data from a large case-control study conducted in Italian areas between 1991 and 1996.

*Received 24 June 1998*
*Revised 9 October 1998*
*Accepted 13 October 1998*

*Correspondence to:* A Tavani

### SUBJECTS AND METHODS

The data were derived from a case-control study of colorectal cancer conducted between June 1991 and 1996 (La Vecchia et al, 1997) in six Italian areas: greater Milan; the provinces of Pordenone and Gorizia; the urban area of Genoa; the province of Forli (northern Italy); the province of Latina (central Italy) and the urban area of Naples (southern Italy). The interviewers were centrally trained and the structured questionnaires were tested for reliability and reproducibility of dietary variables (Franceschi et al, 1993; Decarli et al, 1996). Less than 4% of cases and controls approached for interview refused to participate and the response rates did not vary across hospitals and geographical areas.

Cases of colon cancer comprised 688 men (median age, 62; range, 19 to 74 years) and 537 women (median age, 61; range, 24 to 74 years) with incident, i.e. diagnosed within the year before interview, histologically confirmed cancer, and all had been admitted to the major teaching and general hospitals in the areas under surveillance. Cases of rectal cancer comprised 435 men and 286 women (median age, 62; range, 24 to 74 years) with incident, histologically confirmed cancer. Cancers of the colon and rectum and their anatomical subsites were defined according to the

**Table 1** Distribution of 1225 colon cancer cases and 4154 controls according to selected covariates, Italy, 1991–1996

| | Colon cancer | | Controls | |
|---|---|---|---|---|
| | **Men** | **Women** | **Men** | **Women** |
| Age (years) | | | | |
| < 40 | 26 | 29 | 155 | 192 |
| 40–49 | 57 | 57 | 327 | 405 |
| 50–59 | 174 | 147 | 610 | 634 |
| 60–69 | 320 | 198 | 713 | 643 |
| ≥ 70 | 111 | 106 | 268 | 207 |
| Education (years) | | | | |
| < 7 | 306 | 315 | 1057 | 1219 |
| 7–11 | 210 | 121 | 629 | 527 |
| ≥ 12 | 172 | 101 | 387 | 335 |
| Total alcohol intake (g ethanol day$^{-1}$) | | | | |
| 0 | 98 | 196 | 297 | 876 |
| < 26.9 | 199 | 274 | 609 | 984 |
| ≥ 26.9 | 391 | 67 | 1167 | 221 |
| Total energy intake (kcalories day$^{-1}$)[a] | | | | |
| < 2079 | 130 | 235 | 369 | 1000 |
| 2079 to < 2717 | 233 | 188 | 699 | 672 |
| ≥ 2717 | 325 | 114 | 1005 | 409 |

[a]Approximate tertiles.

International Classification of Diseases, 9th Edition (ICD-9). Colon cancer corresponded to ICD-9 153; ascending colon included ICD-9 153.0, 153.4, 153.5 and 153.6; transverse and descending colon ICD-9 153.1, 153.2 and 153.7; and sigmoid colon to ICD-9 153.3. Rectal cancer included ICD-9 154.0–154.1; rectosigmoid junction corresponded to ICD-9 154.0; and rectum to ICD-9 154.1. Controls comprised 2073 men (median age, 59; range, 19 to 74 years) and 2081 women (median age, 56; range, 20 to 74 years), residing in the same geographical areas and admitted to the same network of hospitals where the cases had been identified, for a wide spectrum of acute conditions unrelated to known or potential risk factors for colorectal cancer. Of these, 23% had traumatic conditions (mostly fractures and sprains), 28% non-traumatic orthopaedic disorders (mostly low back pain and disc disorders), 20% acute surgical conditions (mostly abdominal, such as acute appendicitis or strangulated hernia), and 29% miscellaneous other illnesses (such as eye, ear, nose, throat and dental disorders).

All interviews were conducted in hospital using a structured questionnaire which included information on personal characteristics and habits, anthropometric variables, education and other socio-economic factors, general lifestyle habits, such as smoking, alcohol, coffee consumption, medical history and dietary habits. The section on physical activity included questions on self-reported intensity of activity at work and in leisure time separately. Physical activity was elicited for four specific periods of life: at the ages of 12, 15 to 19, 30 to 39 and 50 to 59 years. For occupational physical activity, patients were asked about their jobs, which were classified into five categories (scores between 1 and 5) corresponding to ‘very heavy’, ‘heavy’, ‘average’, ‘standing’ and ‘mainly sitting’. Physical activity in leisure time was defined on the basis of the number of hours per week of a sport or activity such as walking, gardening, cycling, etc. The scores ranged

**Table 2** Distribution of colon cancer cases and controls, and corresponding odds ratios (OR) with 95% confidence intervals (CI), according to the level of occupational physical activity at various ages, Italy, 1991–1996

| | Men | | | Women[a] | | |
|---|---|---|---|---|---|---|
| | **Cases** | **Controls** | **OR (95% CI)**[b] | **Cases** | **Controls** | **OR (95% CI)**[b] |
| At 15–19 years old | | | | | | |
| 1 (lowest) | 202 | 425 | 1[c] | 188 | 639 | 1[c] |
| 2 | 104 | 266 | 0.89 (0.64–1.23) | 126 | 654 | 0.73 (0.55–0.96) |
| 3 | 173 | 532 | 0.72 (0.54–0.97) | 150 | 487 | 0.91 (0.69–1.21) |
| 4 | 165 | 654 | 0.54 (0.40–0.74) | 73 | 299 | 0.62 (0.44–0.89) |
| 5 (highest) | 44 | 194 | 0.47 (0.31–0.71) | | | |
| Unknown | – | 2 | | – | 2 | |
| $\chi^2$ trend | | | 21.84** | | | 3.89* |
| At 30–39 years old | | | | | | |
| 1 (lowest) | 112 | 243 | 1[c] | 75 | 217 | 1[c] |
| 2 | 155 | 352 | 1.01 (0.75–1.37) | 116 | 465 | 0.65 (0.46–0.93) |
| 3 | 184 | 574 | 0.79 (0.59–1.06) | 245 | 993 | 0.57 (0.41–0.79) |
| 4 | 162 | 588 | 0.71 (0.52–0.97) | 91 | 346 | 0.49 (0.33–0.72) |
| 5 (highest) | 71 | 282 | 0.64 (0.44–0.93) | | | |
| Unknown | 2 | 6 | | – | 7 | |
| $\chi^2$ trend | | | 9.81** | | | 12.56** |
| At 50–59 years old | | | | | | |
| 1 (lowest) | 117 | 234 | 1[c] | 56 | 153 | 1[c] |
| 2 | 148 | 304 | 1.06 (0.78–1.43) | 145 | 562 | 0.69 (0.47–1.00) |
| 3 | 173 | 460 | 0.85 (0.63–1.14) | 186 | 578 | 0.68 (0.46–1.00) |
| 4 | 116 | 406 | 0.68 (0.49–0.95) | 60 | 154 | 0.75 (0.47–1.20) |
| 5 (highest) | 45 | 154 | 0.69 (0.45–1.05) | | | |
| Unknown | 6 | 33 | | 4 | 37 | |
| $\chi^2$ trend | | | 8.59** | | | 1.02 |

[a]The two categories with the highest level of physical activity were combined. [b]Estimates from multiple logistic regression equations, including terms for centre, age, intake of total alcohol and energy, and education. [c]Reference category: *$P < 0.05$, **$P < 0.01$.

**Table 3** Distribution of colon cancer cases in anatomical subsites and corresponding odds ratios (OR) with 95% confidence intervals (CI), according to the level of occupational physical activity at 30–39 years old, Italy 1991–1996[a]

| | Men | | Women | |
|---|---|---|---|---|
| | **No.** | **OR (95% CI)[b]** | **No.** | **OR (95% CI)[b]** |
| Ascending colon | | | | |
| 1 (lowest) | 16 | 1[c] | 13 | 1[c] |
| 2 | 20 | 0.91 (0.45–1.82) | 15 | 0.41 (0.18–0.92) |
| 3 | 26 | 0.78 (0.40–1.52) | 36 | 0.46 (0.23–0.94) |
| 4 (highest) | 42 | 0.79 (0.40–1.53) | 15 | 0.43 (0.19–1.00) |
| $\chi^2$ trend | | 0.59 | | 2.37 |
| Transverse and descending colon | | | | |
| 1 (lowest) | 23 | 1[c] | 13 | 1[c] |
| 2 | 28 | 0.92 (0.51–1.67) | 19 | 0.51 (0.23–1.10) |
| 3 | 32 | 0.76 (0.43–1.37) | 32 | 0.39 (0.19–0.80) |
| 4 (highest) | 27 | 0.46 (0.24–0.87) | 11 | 0.29 (0.12–0.71) |
| $\chi^2$ trend | | 6.54* | | 7.42** |
| Sigmoid colon | | | | |
| 1 (lowest) | 45 | 1[c] | 29 | 1[c] |
| 2 | 58 | 1.02 (0.65–1.57) | 41 | 0.62 (0.36–1.05) |
| 3 | 68 | 0.78 (0.51–1.20) | 100 | 0.71 (0.44–1.15) |
| 4 (highest) | 66 | 0.54 (0.34–0.85) | 32 | 0.58 (0.32–1.03) |
| $\chi^2$ trend | | 9.50** | | 1.91 |

[a]The sum does not add up to the total because of some missing values. The two categories of the highest level of physical activity were combined. [b]Estimates from multiple logistic regression equations, including terms for centre, age, education and intake of total alcohol and energy. [c]Reference category*:* *$P < 0.05$, **$P < 0.01$.

**Table 4** Distribution of rectal cancer cases and corresponding odds ratios (OR) with 95% confidence intervals (CI), according to level of occupational physical activity at 30–39 years old, Italy 1991–1996[a]

| | Men | | Women | |
|---|---|---|---|---|
| | **No.** | **OR (95% CI)[b]** | **No.** | **OR (95% CI)[b]** |
| Rectosigmoid junction | | | | |
| 1 (lowest) | 15 | 1[c] | 13 | 1[c] |
| 2 | 17 | 0.78 (0.37–1.64) | 13 | 0.38 (0.16–0.87) |
| 3 | 13 | 0.42 (0.19–0.93) | 34 | 0.48 (0.23–0.97) |
| 4 (highest) | 41 | 0.85 (0.42–1.72) | 12 | 0.39 (0.16–0.94) |
| $\chi^2$ trend | | 0.14 | | 2.67 |
| Rectum | | | | |
| 1 (lowest) | 39 | 1[c] | 22 | 1[c] |
| 2 | 67 | 1.32 (0.84–2.06) | 44 | 1.03 (0.59–1.81) |
| 3 | 102 | 1.39 (0.91–2.12) | 106 | 0.91 (0.54–1.52) |
| 4 (highest) | 142 | 1.32 (0.86–2.03) | 42 | 0.88 (0.48–1.60) |
| $\chi^2$ trend | | 0.93 | | 0.40 |

[a]The sum does not add up to the total because of some missing values. The two categories of highest physical activity were combined. [b]Estimates from multiple logistic regression equations, including terms for centre, age, education and intake of total alcohol and energy. [c]Reference category.

between 1 and 4, corresponding to < 2, 2–4, 5–7 and > 7 h of physical activity per week. No information was available on intensity or amount of activity. For women, the scores of the two highest levels of either occupational or leisure-time physical activity were combined, as there were too few women in the categories with the highest activity.

### Data analysis

Odds ratios (OR) of colon cancer, and the corresponding 95% confidence intervals (CI) for increasing levels of occupational and leisure-time physical activity at various ages were derived using unconditional multiple logistic regression, fitted by the method of maximum likelihood (Breslow and Day, 1980) in men and women separately. Two models were considered: the first (Model A) including terms for geographical area, quinquennia of age and total intake of alcohol and energy (not shown); and the second (Model B) with further terms for education.

### RESULTS

Table 1 gives the distribution of colon cancer cases and the comparison group according to age, education and alcohol and energy intake in men and women. The cancer patients were more

*© Cancer Research Campaign 1999*

educated and reported higher energy intakes than the controls. Table 2 shows the distribution of colon cancer cases and controls with corresponding multivariate ORs (estimated by Model B) for men and women, in relation to their occupational levels of physical activity at different ages. Compared with men in the lowest level of occupational physical activity, the ORs (Model A) of colon cancer for men in the highest level were 0.39, 0.51 and 0.57 (statistically significant), respectively, at 15–19, 30–39 and 50–59 years old, with significant trends in risk. Similarly, in women, the corresponding ORs were 0.54, 0.44 and 0.66. The inverse association was weaker, but still significant after further allowance for education, and the ORs for the highest level of activity compared with the lowest in subsequent age groups were 0.47, 0.64 and 0.69 in men, and 0.62, 0.49 and 0.75 in women, with significant trends in risk except for women at 50–59 years old. No consistent association was found between leisure-time physical activity and colon cancer risk for either sex at the same ages (not shown).

A lifelong index of occupational physical activity was also computed, combining the information from various ages considered in subjects aged 50 years or older. For males, the multivariate ORs (Model B) for increasing quintiles of activity compared with the lowest were 0.95, 0.71, 0.83 and 0.39 ($\chi^2$ trend = 18.68, $P < 0.01$); the corresponding values in women were 0.77, 0.73, 0.82 and 0.52 ($\chi^2$ trend = 6.14, $P < 0.05$).

The risk of colon cancer for each level of occupational physical activity at age 30 to 39 years old in separate strata of selected covariates was not significantly heterogeneous across strata for age at diagnosis, education, body mass index and intake of alcohol, total energy, vegetables, fibre or starch.

Table 3 shows the risk of cancer in anatomical subsites of the colon for levels of occupational physical activity at 30 to 39 years old. The protection appeared consistent in the colon subsites considered; the ORs for the highest level of activity for men and women were, respectively, 0.79 and 0.43 for the ascending colon, 0.46 and 0.29 for the transverse and descending colon, and 0.54 and 0.58 for the sigmoid colon.

The relation between risk of rectal cancer and occupational physical activity at 30 to 39 years old is reported in Table 4. There was no consistent association between physical activity and cancers of the rectosigmoid junction or rectum in either sex. The ORs were somewhat below unity for the rectosigmoid junction, which presents problems of misclassification of the sigma (Doll, 1980). Similarly, no association was found with physical activity at work and in leisure time at the ages of 12, 15 to 19 and 50 to 59 years old for either sex (not shown).

### DISCUSSION

Several biological mechanisms have been proposed to explain the influence of exercise on colon carcinogenesis, possibly involving multiple but, in some cases, overlapping pathways (Macfarlane and Lowenfels, 1994; Potter, 1995; Colditz et al, 1997). Physical activity may increase colonic motility, which in turn is associated with reduced colon cancer risk (Cummings et al, 1992; Potter, 1995), possibly because of the shorter contact between the colonic mucosa and possible carcinogens. This theory, however, lacks real experimental or epidemiological evidence (Macfarlane and Lowenfels, 1994). However, physical activity also reduces insulin resistance, thus avoiding colon cancer stimulation possibly linked to hyperglycaemia and hyperinsulinaemia (Macfarlane and Lowenfels, 1994; Colditz et al, 1997), as supported by the finding that people with a high level of physical activity have a lower incidence of non-insulin-dependent diabetes mellitus (Helmirich et al, 1991). Other possible mechanisms include the interaction with body mass and body fat composition, changes in serum cholesterol and bile acid metabolism, or, in women, changes in levels and availability of sex hormones (Macfarlane and Lowenfels, 1994; Potter, 1995). Exercise can also raise the production of some prostaglandins that, in turn, may influence colon cancer risk (Demers et al, 1981; Colditz, et al, 1997), and the activation of the immune system response has also been postulated (Simon, 1984).

The lack of association between colon cancer risk and leisure-time physical activity might depend on its low level and frequency among Italian men and particularly among women. Moreover, it is usually shorter lasting, more often restricted to younger people, is more difficult to define and quantify accurately than occupational activity, and, in Italy, mostly involves people in the highest social classes, which is a correlate of colon cancer (Potter et al, 1993).

Some of the associations with low occupational physical activity might also be explained in terms of social class correlates. Allowance for educational level somewhat attenuated the inverse relationship of occupational physical activity with colon cancer risk, but the general pattern of protection clearly persisted. Adjustment for education may, however, constitute an overadjustment, because educational level is largely related to physical activity at work. The potential confounding effect of several other covariates was allowed for in the analysis, but there was no material modification in the pattern of risk estimates.

There are problems of reliability and validity in the assessment of physical activity because we used a subjective score with no quantification of total energy expenditure. However, the similar interview settings provide reassurance against potential information bias (D'Avanzo et al, 1997) and there is no reason to assume different recall on the basis of the disease status, as the inverse association between colon cancer risk and physical activity is not a matter of public knowledge in Italy. This study was not population-based, but cases were identified in the major teaching and general hospitals of the study area, the participation of cases and controls was almost complete, and we excluded from the control group patients admitted to hospital for chronic conditions or digestive tract diseases. Exclusion of controls with orthopaedic (including traumatic conditions) from the analysis did not modify the risk estimates. Further reassurance that the medical conditions of controls did not influence the results comes from the consistency of the protective effect of physical activity across different ages, even from long before the diagnosis that led to hospital admission.

The attributable risk of colon cancer in this population, under the assumption that the physical activity of the entire population could be increased to the two highest levels, was 15% (95% CI, 4–26%) in men and 17% (95% CI, 1–36%) in women (Mezzetti et al, 1996), which was similar to that reported in a North American study (Slattery et al, 1997). The finding that a high level of occupational physical activity across ages confers some protection against colon cancer is particularly important in view of the benefits of physical activity on other chronic diseases too, such as cancer at certain other sites and cardiovascular diseases (La Vecchia and Negri, 1992).

### ACKNOWLEDGEMENTS

This work was conducted with the contribution of the Italian Association for Cancer Research, Milan, and of the 'Europe

© Cancer Research Campaign 1999

against Cancer Programme' of the Commission of the European Communities. The authors thank Mrs J Baggott, MP Bonifacino and the GA Pfeiffer Memorial Library staff for editorial assistance.

### REFERENCES

Breslow NE and Day NE (1980) *Statistical Methods in Cancer Research, Vol. 1, The analysis of case-control studies*. IARC Scientific Publications, **32**

Colditz GA, Cannuscio CC and Frazier AL (1997) Physical activity and reduced risk of colon cancer: implications for prevention. *Cancer Causes Control* **8**: 649–667

Cummings JH, Bingham SA, Heaton KW and Eastwood MA (1992) Fecal weight, colon cancer risk, and dietary intake of nonstarch polysaccharides (dietary fiber). *Gastroenterology* **103**: 1783–1789

D'Avanzo B, La Vecchia C, Katsouyanni K, Negri E and Trichopoulos D (1997) Hospital and population controls: an assessment of and reproducibility of food frequency data. *Eur J Cancer Prevention* **6**: 288–293

Decarli A, Franceschi S, Ferraroni M, Gnagnarella P, Parpinel MT, La Vecchia C, Negri E, Salvini S, Falcini F and Giacosa A (1996) Validation of a food frequency questionnaire to assess dietary intakes in cancer studies in Italy: results for specific nutrients. *Ann Epidemiol* **6**: 110–118

Demers LM, Harrison TS, Halbert DR and Santen RJ (1981) Effect of prolonged exercise on plasma prostaglandin levels. *Prostaglandins Med* **6**: 413–418

Doll R (1980) General epidemiologic considerations in etiology of colorectal cancer. In *Colorectal Cancer: Prevention, Epidemiology and Screening. Progress in Cancer Research and Therapy, Vol. 3*. Winawer S, Schottenfeld D, Sherlock P (eds), pp. 3–12. Raven Press: New York

Franceschi S, Negri E, Salvini S, Decarli A, Ferraroni M, Filiberti R, Giacosa A, Talamini R, Nanni O, Panarello G and La Vecchia C (1993) Reproducibility of an Italian food frequency questionnaire for cancer studies: results for specific food items. *Eur J Cancer* **29A**: 2298–2305

Helmrich SP, Ragland DR, Leung RW and Paffenbarger RS Jr (1991) Physical activity and reduced occurrence of non-insulin-dependent diabetes mellitus. *N Engl J Med* **325**: 147–152

La Vecchia C and Negri E (1992) Public education on diet and cancer: calories, weight and exercise. In *Public Education on Diet and Cancer*. Benito E, Giacosa A, Hill MJ (eds), pp. 91–100. Kluwer: Dordrecht

La Vecchia C, Negri E, Franceschi S, Conti E, Montella M, Decarli A and Filiberti R (1997) Aspirin and colorectal cancer. *Br J Cancer* **76**: 675–677

Lee I-M, Paffenbarger RS Jr and Hsieh C-C (1991) Physical activity and risk of developing colorectal cancer among college alumni. *J Natl Cancer Inst* **83**: 1324–1329

Longnecker MP, Gerhardsson de Verdier M, Frumkin H and Carpenter C (1995) A case-control study of physical activity in relation to risk of cancer of the right colon and rectum in men. *Int J Epidemiol* **24**: 42–50

Macfarlane GJ and Lowenfels AB (1994) Physical activity and colon cancer. *Eur J Cancer Prev* **3**: 393–398

Marcus PM, Newcomb PA and Storer BA (1994) Early adulthood physical activity and colon cancer risk among Wisconsin women. *Cancer Epidemiol Biomarkers Prev* **3**: 641–644

Mezzetti M, Ferraroni M, Decarli A, La Vecchia C and Benichou J (1996) Software for attributable risk and confidence interval estimation in case-control studies. *Comput Biomed Res* **29**: 63–75

Potter JD (1995) Risk factors for colon neoplasia. Epidemiology and biology. *Eur J Cancer* **31A**: 1033–1038

Potter JD, Slattery ML, Bostick RM and Gapstur SM (1993) Colon cancer: a review of the epidemiology. *Epidemiol Rev* **15**: 499–545

Simon HB (1984) The immunology of exercise. A brief review. *JAMA* **252**: 2735–2738

Slattery ML, Edwards SL, Ma K-N, Friedman GD and Potter JD (1997) Physical activity and colon cancer: a public health perspective. *Ann Epidemiol* **7**: 137–145

*© Cancer Research Campaign 1999*
